# Supplementary material for: Compensating Complete Loss of Signal Recognition Particle During Co-translational Protein Targeting by the Translation Speed and Accuracy
Source: Front Microbiol. 2021 Jul 9;12:690286. doi: 10.3389/fmicb.2021.690286 (PMC8299109; doi:10.3389/fmicb.2021.690286)
Supplement: Supplementary file 1 [file Data_Sheet_1.pdf]

Supplementary Material

Supplementary Figures and Tables

Supplementary Table 1A. Strains used in this study.

Strain	Genotype	Source or Reference
MG1655	K-12 F^- λ^- rph-1	(Blattner et al., 1997)
HDB51	WAM113 *secB*$^+$ *zic-4901*::Tn10	(Lee and Bernstein, 2001)
MY1410	MG1655 *ffh*::Cm^r, pKDFB	(Zhao et al., 2021)
MY1901	MG1655 *ffh*:: Cm^r, C3453283T	This study
MY1901F	MY1901, pTrc99K-Ffh	This study
MY1901FS	MY1901F, T3453283C	This study
MY1901Δ*cat*	MY1901, Δ*cat*	This study
MG1655Δ*lacZ*	MG1655, Δ*lacZ*	(Zhao et al., 2021)
MY1901Δ*lacZ*Δ*cat*	MY1901, Δ*lacZ*Δ*cat*	This study

Supplementary Table 1B. Plasmids used in this study.

Plasmid	Relevant genotype	Source or Reference
pTrc99K-Ffh	pTrc99K, P_{trc}-*ffh*, Km^r	(Zhao et al., 2021)
pKUT15-fusA-lacZα	OriSC101 lacIq P_{lac}-*fusA-lacZα*, P_{tet}-lacZω, Gm^r	(Dai et al., 2018)
pKUT15-lacZα	pKUT15, P_{lac}-*lacZα*, P_{tet}-*lacZω*	(Zhao et al., 2021)
pKUT15-msbA-lacZα	pKUT15, P_{lac}-*msbA-lacZα*, P_{tet}-*lacZω*	(Zhao et al., 2021)
pLacZ	pTrc99K, P_{trc}-*lacZ*	This study
$pLacZ_{835UAA}$	pLacZ, UAA mutant	(O'Connor et al., 1992)
$pLacZ_{12\text{-}6UAG}$	pLacZ, UAG mutant	(O'Connor et al., 1992)
$pLacZ_{34\text{-}11UGA}$	pLacZ, UGA mutant	(O'Connor et al., 1992)
$pLacZ_{lac10(-)}$	pLacZ, -1 frameshift	(O'Connor et al., 1992)
$placZ_{lac7(+)}$	pLacZ, +1 frameshift	(O'Connor et al., 1992)
pRed_Cas9_ΔpoxB300	OrirepA101, exo, bet, gam, arabinose operon, Cas9, gRNA, Ap^r	(Zhao et al., 2016)
pGFP	pTrc99K, P_{trc}-gfp_{AUG}	This study
$pGFP_{GUG}$	pTrc99K, P_{trc}-gfp_{GUG}	This study
$pGFP_{UUG}$	pTrc99K, P_{trc}-gfp_{UUG}	This study
$pGFP_{AUA}$	pTrc99K, P_{trc}-gfp_{AUA}	This study
$pGFP_{AUU}$	pTrc99K, P_{trc}-gfp_{AUU}	This study
$pGFP_{AUG}metY_{CAU}$	pGFP, expressing $tRNA^{fMet}$ with CAU anticodon	This study
$pGFP_{UAG}metY_{CUA}$	$pGFP_{AUG}metY_{CAU}$, AUG initiation codon mutated to UAG and anticodon of metY was changed from CAU to CUA	This study
$pGFP_{CAC}metY_{GUG}$	$pGFP_{AUG}metY_{CAU}$, AUG initiation codon mutated to CAC and anticodon of metY was changed from CAU to GUG	This study
$pGFP_{UAC}metY_{GUA}$	$pGFP_{AUG}metY_{CAU}$, AUG initiation codon mutated to UAC and anticodon of metY was changed from CAU to	This study

	GUA	
Cas9-S10 SD	pRed_Cas9_ΔpoxB300, homologous arms of SD of S10	This study
Cas9-lacZ	pRed_Cas9_ΔpoxB300, homologous arms of lacZ	(Zhao et al., 2021)
Cas9-cat	pRed_Cas9_ΔpoxB300, homologous arms of cat	(Zhao et al., 2021)
p15A-birA	Orip15A, lacIq P_{tac}-*birA*, Gm^r	This study
pJH29-EspP-PSBT	pJH29, P_{tac}-*espP*, PSBT-FLAG, Cm^r	(Zhang and Shan, 2012)
pJH29-EspP-Avi	pJH29, P_{tac}-*espP*, Avi-FLAG, Cm^r	This study
pJH29-FtsQ-Avi	pJH29, P_{tac}-*ftsQ*, Avi-FLAG, Cm^r	This study
pJH29-LacZ-Avi	pJH29, P_{tac}-*lacZ*, Avi-FLAG, Cm^r	This study
pJH30-EspP-PSBT	pJH29, *lac* operon replaced with *araC* operon, Ap^r	(Zhao et al., 2021)
pJH30-SD-GFP	pJH30, P_{araBAD}-GFP, wild-type SD, Ap^r	This study
pJH30-SD*-GFP	pJH30, P_{araBAD}-GFP, mutant SD, Ap^r	This study
pJH31-SD-GFP	pJH30, P_{araBAD} replaced with $P_{S10,}$ wild-type SD Ap^r	This study
pJH31-SD*-GFP	pJH31, P_{S10}-GFP, mutant SD, Ap^r	This study

Supplementary Table 1C. Primers used in this study.

Primer	Sequence (5'-> 3')	Use
pTrc99K-F	GATCCTCTAGAGTCGACCT	Cloning of the vector bone of plasmid pTrc99K
pTrc99K-R	GAGCTCGAATTCCATGGTCT	
GFP-F	ACAGACCATGGAATTCGAGCTCATGAGTA AAGGAGAAGAACTTTTC	Cloning of *gfp* segment
GFP-R	TGCAGGTCGACTCTAGAGGATCCTATTTG TATAGTTCATCCATG	
GFP_{GUG}-F	ACAGACCATGGAATTCGAGCTCGTGAGT AAAGGAGAAGAACTTTTC	Cloning of gfp_{GUG} with segment
GFP_{UUG}-F	ACAGACCATGGAATTCGAGCTCTTGAGT AAAGGAGAAGAACTTTTC	Cloning of gfp_{UUG} with segment
GFP_{AUA}-F	ACAGACCATGGAATTCGAGCTCATAAGT AAAGGAGAAGAACTTTTC	Cloning of gfp_{AUA} with segment
GFP_{AUU}-F	ACAGACCATGGAATTCGAGCTCATTAGT AAAGGAGAAGAACTTTTC	Cloning of gfp_{AUU} with segment
MetY-V-R	GTGAAAACCTCTGACACATG	Cloning of the vector bone of plasmid pGFP with primer pTrc99K-F
$MetY_{CAU}$-F	CTGCATGTGTCAGAGGTTTTCACTGGAT CACTATAATGCCTGC	Construction of plamid $pGFP_{AUG}metY_{CAU}$
$MetY_{CAU}$-R	CTATCATGCCATACCGCGAAAGGAGCC CAGTTATTCTGTAGTC	
MetY-V-F	CCTTTCGCGGTATGGCATG	
$MetY_{CUA}$-F	GTCGGGCTCTAAACCCGAAGATCGT	Construction of plamid $pGFP_{UAG}metY_{CUA}$
$MetY_{CUA}$-R	ACGATCTTCGGGTTTAGAGCCCGACG	
GFP_{UAG}-F	ACAGACCATGGAATTCGAGCTCTAGAGTAA AGGAGAAGAACTTTTC	
$MetY_{GUG}$-F	GTCGGGCTGTGAACCCGAAGATCGT	Construction of plamid $pGFP_{CAC}metY_{GUG}$
$MetY_{GUG}$-R	ACGATCTTCGGGTTCAGAGCCCGACG	
GFP_{CAC}-F	ACAGACCATGGAATTCGAGCTCCACAGTAA AGGAGAAGAACTTTTC	
$MetY_{GUA}$-F	GTCGGGCTGTAAACCCGAAGATCGT	Construction of plamid $pGFP_{UAC}metY_{GUA}$
$MetY_{GUA}$-R	ACGATCTTCGGGTTTAGAGCCCGACG	
GFP_{UAC}-F	ACAGACCATGGAATTCGAGCTCTACAGTAA AGGAGAAGAACTTTTC	
V1-cas9-F	TGAATGGAAGCTTGGATTCTC	Cloning of the vector bone 1 of plasmid Cas9-S10 SD
V1-S10-R	TATCCGCCTGAAAGCGTTTGGCTAAGATCTG ACTCCATAAC	

V2-S10-F	CAAACGCTTTCAGGCGGATAGTTTTAGAGCTAGAAATAGCAAG	Cloning of the vector bone 2 of plasmid Cas9-S10 SD
V2-cas9-R	ACAGGCCCATGGATTCTTCG	
S10-UP-F	CGAAGAATCCATGGGCCTGTTTAACCCAGGCTGATCTGC	Cloning of the upstream of the mutated site
S10-UP-R	CTGGTCTCATGCAGAACCAAAGAATACGTATCC	
S10-DN-F	TGGTTCTGCATGAGACCAGAGCTCCAATTAT	Cloning of the downstream of the mutated site
S10-DN-R	AGAATCCAAGCTTCCATTCAAAGAGAAAGCCGGTTTAAGAG	
p15-V-F	CCCAGGCATCAAATAAAACG	Cloning of the vector bone of plasmid p15A-birA
p15-V-R	GATCCGAATTCCTGCAGTTG	
birA-F	TAACAACTGCAGGAATTCGGATCATGAAGGATAACACCGTGCC	Cloning of the *birA* segment
birA-R	TTTCGTTTTATTTGATGCCTGGGTTATTTTTCTGCACTACGCAG	
29-V-F	TTCCGGCTTGAACGACATCTTCGAGGCCCAGAAGATCGAGTGGCACGAGGGTTCTGGTGACTACAAAGACGATGACGAC	Cloning of the vector bone of plasmid pJH29-EspP-Avi from pJH29-EspP-PSBT
29-V-R	ATTAGTCCGCCAGTTCCAC	
espP-F	TATGTGGAACTGGCGGACTAATATGAATAAAATATACTCTC	Cloning of the *espP* segment
espP-R	CGAAGATGTCGTTCAAGCCGGAACCGATATAGTCAGCAGTATTATTC	
FtsQ-Avi-V-F	CTTCGAGGCCCAGAAGATCGAGTGGCACGAGGGTTCTGGTGACTACAAAGACGATGACGAC	Cloning of the vector bone of plasmid pJH29-FtsQ-Avi from pJH29-EspP-Avi with primer 29-V-R
FtsQ-Avi-F	TATGTGGAACTGGCGGActaatATGTCGCAGGCTGCTCTG	Cloning of the *ftsQ-Avi* tag segment
FtsQ-Avi-R	ACTCGATCTTCTGGGCCTCGAAGATGTCGTTCAAGCCTCTAGATTGTTGTTCTGCCTGTGCCT	
LacZ-Avi-V-F	TTCCGGCTTGAACGACATCT	Cloning of the vector bone of plasmid pJH29-LacZ-Avi from pJH29-EspP-Avi with primer 29-V-R
LacZ-Avi-F	ATGTGGAACTGGCGGACTAATATGACCATGATTACGGATTCAC	Cloning of the *lacZ-Avi* tag segment
LacZ-Avi-R	AAGATGTCGTTCAAGCCGGAACCTTTTTGACACCAGACCAACTG	
pJH30-F	ATCAGTAAGTTGGCAGCATC	Cloning of the vector bone of plasmid pJH30
pJH30-R	ATGGAGAAACAGTAGAGAGTTG	
S10 leader-F	CAACTCTCTACTGTTTCTCCATGGCTACCTAACAATGCTCC	Cloning of the S10 leader containing SD from MG1655 genome (wild-type SD) or MY1901 genome (mutant SD)
S10 leader-R	AAAGTTCTTCTCCTTTACTCATCGGATACGGATTCTTTGGTTCTG	
GFP-F	ATGAGTAAAGGAGAAGAACTTTTC	Cloning of the *gfp* segment
GFP-R	TGATGCTGCCAACTTACTGATCTATTTGTATAGTTCATCCATG	
pJH31-R	CGCGGAACCCCTATTTGTT	Cloning of the vector bone of plasmid pJH31 with primer pJH30-F
P_{S10}-F	AATAAACAAATAGGGGTTCCGCGGAGCGTGTCAAAAATGCACTG	Cloning of the P_{S10} promoter and S10 leader containing SD from MG1655 genome or MY1901 genome

Supplementary Table 2. Summary of single nucleotide variants (SNVs), and insertions and deletions (INDELs). The strain MY1901 shared common mutations with the original strain MG1655 in the laboratory, except the mutation located in the Shine-Dalgarno (SD) sequence of ribosome S10 operon. The evolved strain MY1901 by sub-culturing showed new mutations.

**Strain**	**Mutation site**	**Type**	**Chrom Start**	**chrom END**	**Ref**	**Obs**	**Relative to original strain**
MY1901	*fhuA*	SNV	168123	168123	A	C	
	ybhJ	SNV	803662	803662	C	A	
	mntP	SNV	1905761	1905761	G	A	
	yjbI	SNV	4251856	4251856	T	C	
	gatC	INDEL	2173363	2173364	CC	-	
	glpR	INDEL	3560455	3560455	-	G	
	Promoter yagEp3	SNV	282234	282234	G	T	
	A repetitive extragenic palindrome (REP) element downstream of yjcO	INDEL	4296381	4296381	-	GC	
	Shine-Dalgarno (SD) sequence of ribosome S10 operon	SNV	3453283	3453283	C	T	unique
MY1901 (evolved)	*rpsJ*	SNV	3453146	3453146	A	C	
	DnaA	SNV	3882819	3882819	G	T	
	rpoB	SNV	4182952	4182952	G	A	
	rpoC	SNV	4186367	4186367	C	A	

Ref = reference allele; Obs = observed allele.

Supplementary Table 3. Growth rate and the time cost of initiation steps (T_{init}) of cells under different nutrient conditions. Growth rates were calculated from the corresponding growth curves that were shown in Supplementary Figure S4. All values are expressed as the mean ± standard deviation of the mean of samples. The initiation time (T_{init}) was calculated as described in the Materials and Methods section.

**Strain**	**Nutrient condition**	**Growth rate (h^{-1})**	**T_{int}**
MG1655Δ*lacZ*	Glucose + cAA<br>0.2% glucose + 0.2% casamino acids	1.10 ± 0.04	10.0±0.2 (FusA)
			9.6±0.2 (MsbA)
MG1655Δ*lacZ*	Glycerol + NH4Cl<br>0.2% glycerol + 10 mM NH_4Cl	0.55± 0.03	9.6±0.2 (FusA)
			9.1±0.2(MsbA)
MY1905Δ*lacZ*Δ*cat*	Glucose + cAA<br>0.2% glucose + 0.2% casamino acids	0.55± 0.02	11.7±0.3 (FusA)
			10.2±0.2(MsbA)

Supplementary Table 4. The translation rates of FusA-LacZ and MsbA-LacZ under different growth rates. The translation rate equals translation rate (aa s^{-1}) /336 (proteins $mRNA^{-1}$ s^{-1}) (Zarai et al., 2014), as the average length of proteins in *E. coli* is 336 amino acids (Gong et al., 2008). The elongation rate equals elongation rate (aa s^{-1}) /11 (sites s^{-1}) (Zarai et al., 2014) , as each ribosome occupies about 11 residues in *E. coli* (10). All values are expressed as the mean ± standard deviation of the mean of samples.

**Strain**	**Growth rate (h^{-1})**	**Translation rate (proteins $mRNA^{-1}$ s^{-1})**	**Elongation rate (sites s^{-1})**	**Initiation rate (sites s^{-1})**
MG1655Δ*lacZ*	1.10 ± 0.04	(FusA) 0.047 ±0.0003	1.47±0.01	0.049±0.0003
		(MsbA) 0.045 ±0.0003	1.40±0.01	0.047±0.0003
MG1655Δ*lacZ*	0.55± 0.03	(FusA) 0.040±0.001	1.24±0.04	0.042±0.001
		(MsbA) 0.038±0.001	1.15±0.03	0.039±0.001
MY1901Δ*lacZ*Δ*cat*	0.55± 0.02	(FusA) 0.038±0.001	1.22±0.05	0.039±0.001
		(MsbA) 0.032±0.001	1.00±0.03	0.033±0.001

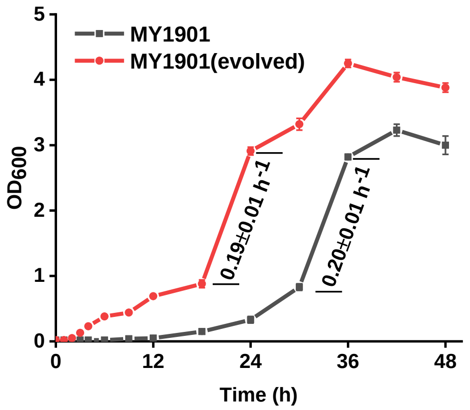

Supplementary Figure 1 Growth curves of strains MY1901 and its evolved strain. Solid curves are the mean of three independent measurements and error bars represent the standard deviation of the mean of samples. All growth rates shown represent the mean ± standard deviation of three independent experiments.

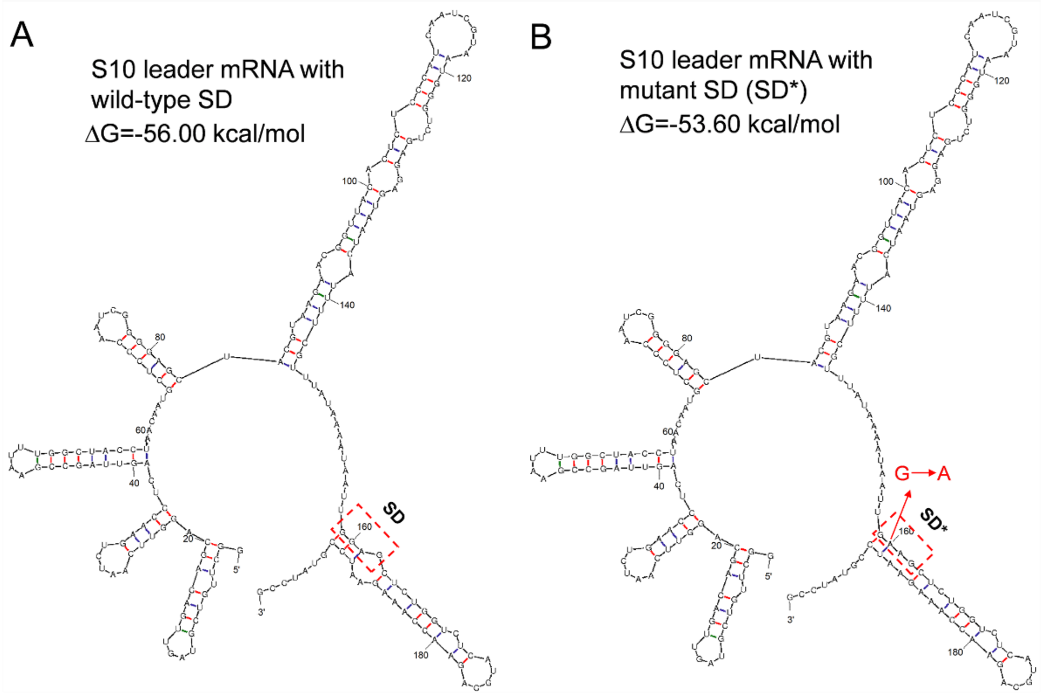

Supplementary Figure 2 Predicted mRNA structures of the S10 leader. **(A)** RNA folding predicted hairpin structure of S10 leader with wild-type SD and the minimum free energy (MFE) of the thermodynamic ensemble (ΔG). **(B)** RNA folding predicted hairpin structure of S10 leader with mutant SD (SD*) and the MFE and the ΔG. The boxed region corresponds to the SD sequence.

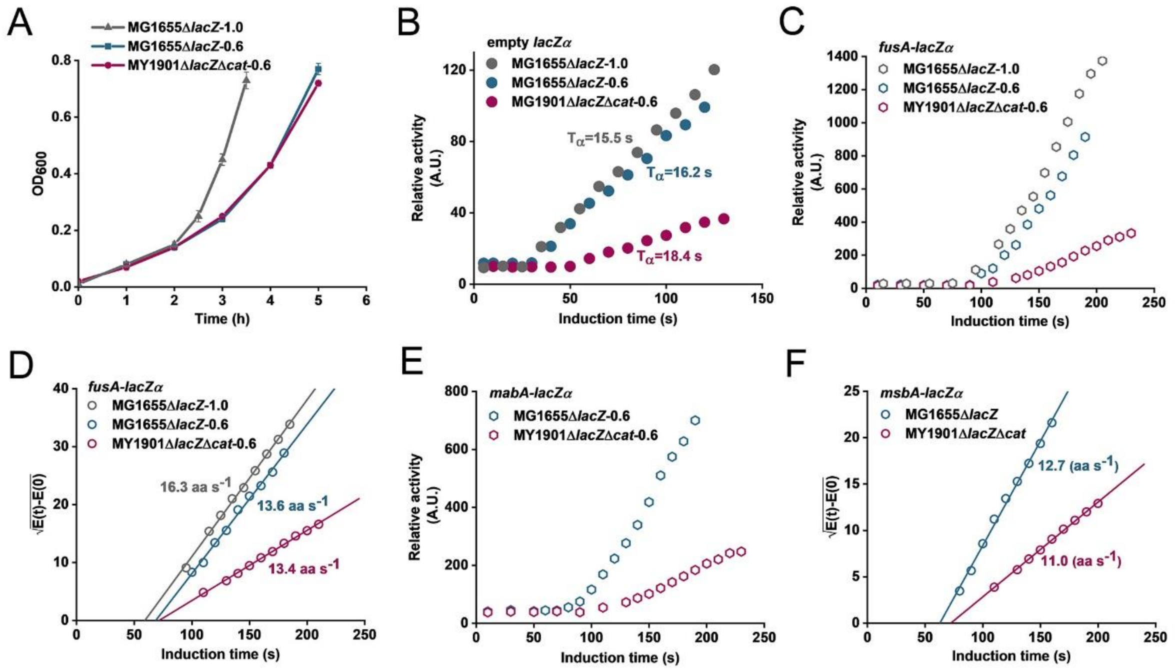

Supplementary Figure 3 Measurement of translational elongation rates of wild-type and suppressor cells. **(A)** Growth curves of strains in different MOPS medium. The growth rates of MG1655Δ*lacZ* and MY1901Δ*lacZ*Δ*cat* grown in the Glucose + cAA medium were approximately 1.0 h^{-1} and 0.6 h^{-1}, respectively. The growth rate of MG1655Δ*lacZ* grown in Glycerol + NH_4Cl medium was approximately 0.6 h^{-1}(Supplementary Table S2). Solid curves are the mean of three independent biological replicates, and error bars represent the standard deviation value. **(B)** Calibration of the time cost of initiation steps by measuring the induction kinetics of the empty LacZα fragment. **(C)** The induction curves of the LacZα fused protein FusA-LacZα. **(D)** The Schleif plot of the FusA-LacZα protein was plotted against the induction time. **(E)** The induction curves of the LacZα fused protein MsbA-LacZα. **(F)** The Schleif plot of the MsbA-LacZα protein was plotted against the induction time. Schleif plots were repeated three times with one typical result shown here.

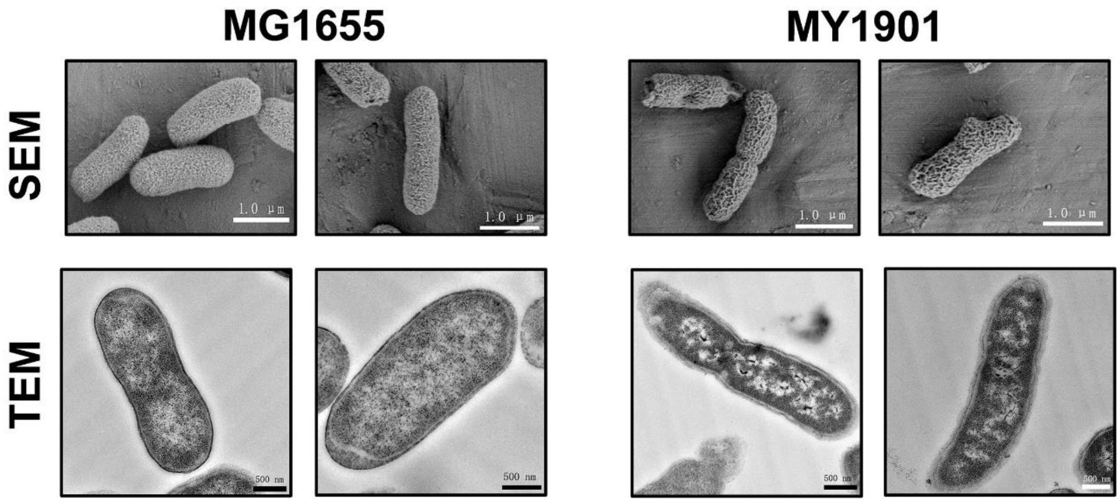

Supplementary Figure 4 Scanning electron microscopy (SEM) and transmission electron microscopy (TEM) analysis of the wild-type strain MG1655 and the suppressor strain MY1901. For SEM, the scale bar is 1.0 μm. For TEM, the scale bar is 500 nM.

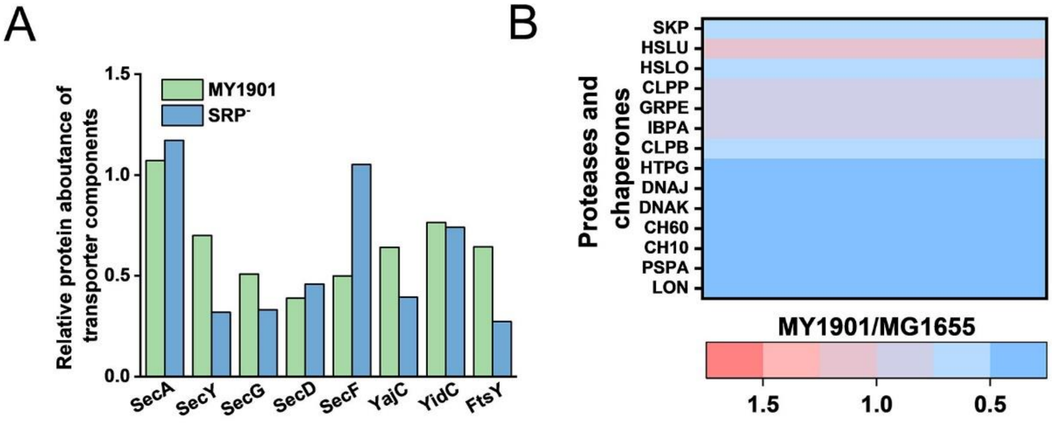

Supplementary Figure 5 Fold changes in the expression of the components of transport machinery **(A)** and the heat shock response-related proteins **(B)** in strain MY1901relative to that in strain MG1655 (**Supplementary Data Set 1B** and **E**).

REFERENCES

Blattner, F.R., Plunkett, G., 3rd, Bloch, C.A., Perna, N.T., Burland, V., Riley, M., Collado-Vides, J., Glasner, J.D., Rode, C.K., Mayhew, G.F., *et al.* (1997). The complete genome sequence of Escherichia coli K-12. *Science* 277, 1453-1462.

Dai, X., Zhu, M., Warren, M., Balakrishnan, R., Okano, H., Williamson, J.R., Fredrick, K., and Hwa, T. (2018). Slowdown of Translational Elongation in Escherichia coli under Hyperosmotic Stress. *mBio* 9, e02375-02317.

Gong, X., Fan, S., Bilderbeck, A., Li, M., Pang, H., and Tao, S. (2008). Comparative analysis of essential genes and nonessential genes in Escherichia coli K12. *Mol Genet Genomics* 279, 87-94.

Lee, H.C., and Bernstein, H.D. (2001). The targeting pathway of Escherichia coli presecretory and integral membrane proteins is specified by the hydrophobicity of the targeting signal. *Proc Natl Acad Sci U S A* 98, 3471-3476.

O'Connor, M., Goringer, H., and Dahlberg, A.E. (1992). A ribosomal ambiguity mutation in the 530 loop of E. coli 16S rRNA. *Nucleic Acids Research* 20, 4221-4227.

Zarai, Y., Margaliot, M., and Tuller, T. (2014). Maximizing Protein Translation Rate in the Ribosome Flow Model: The Homogeneous Case. *IEEE/ACM Trans Comput Biol Bioinform* 11, 1184-1195.

Zhang, D., and Shan, S.O. (2012). Translation elongation regulates substrate selection by the signal recognition particle. *J Biol Chem* 287, 7652-7660.

Zhao, D., Yuan, S., Xiong, B., Sun, H., Ye, L., Li, J., Zhang, X., and Bi, C. (2016). Development of a fast and easy method for Escherichia coli genome editing with CRISPR/Cas9. *Microb Cell Fact* 15, 205.

Zhao, L., Cui, Y., Fu, G., Xu, Z., Liao, X., and Zhang, D. (2021). Signal Recognition Particle Suppressor Screening Reveals the Regulation of Membrane Protein Targeting by the Translation Rate. *mBio* 12, e02373-02320.
